# Supplementary material for: Allosteric inhibition of CRISPR-Cas9 by bacteriophage-derived peptides
Source: Genome Biol. 2020 Feb 26;21:51. doi: 10.1186/s13059-020-01956-x (PMC7045643; doi:10.1186/s13059-020-01956-x)
Supplement: Supplementary file 1 — Figure S1. Comparison of the in vitro activity of M13 and f1 phage G8PPD. Figure S2. MS analyses of the interface between SpCas9 and M13 G8PPD. Figure S3. Construction and purification of SpCas9 K1158 and K1176 mutants. Figure S4. Profile of SpCas9-induced mutations in the absence and presence of G8PPD. Figure S5. G8PPD peptides derived from inoviridae bacteriophages. Figure S6. NGS analyses of the effects of G8P pre-incubation on NmCas9 and SaCas9 in HEK293 cells. Figure S7. NGS analyses of the effects of G8Ps on the specificity of SpCas9 at HBB site in HEK293 cells. Figure S8. Cytotoxicity of CRISPR inhibitors. Figure S9. ChIP-qPCR analyses of the effects of M13 G8Ps on Cas9 binding at AAVS1 on target and pre-determined off-target sites in Hela cells. [file 13059_2020_1956_MOESM1_ESM.docx]

**Additional file 1: Figure S1-S5**

**TABLE OF CONTENT**

1. **Figure S1** Comparison of the *in vitro* activity of M13 and f1 phage G8P_PD_.
2. **Figure S2** MS analyses of the interface between SpCas9 and M13 G8P_PD_.
3. **Figure S3** Construction and purification of SpCas9 K1158 and K1176 mutants.
4. **Figure S4** Profile of SpCas9-induced mutations in the absence and presence of G8P_PD_.
5. **Figure S5** G8P_PD_ peptides derived from inoviridae bacteriophages.
6. **Figure S6** NGS analyses of the effects of G8P pre-incubation on NmCas9 and SaCas9 in HEK293 cells.
7. **Figure S7** NGS analyses of the effects of G8Ps on the specificity of SpCas9 at *HBB* site in HEK293 cells.
8. **Figure S8** Cytotoxicity of CRISPR inhibitors.
9. **Figure S9** ChIP-qPCR analyses of the effects of M13 G8Ps on Cas9 binding at *AAVS1* on target and pre-determined off-target sites in Hela cells.


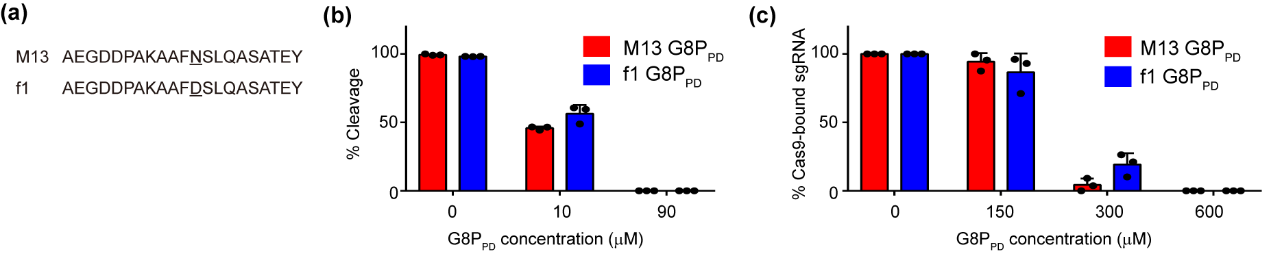


**Figure S1** Comparison of the *in vitro* activity of M13 and f1 phage G8P_PD_. (**a)** Sequence alignment of M13 and f1 G8P_PD_. (**b**) The *in vitro* Cas9-inhibiting activity of M13 and f1 G8P_PD_. M13 and f1 G8P_PD_ of 100 μM were incubated with 50 nM of Cas9 proteins prior to the addition of 50 nM of sgRNA. (c) Inhibition of Cas9-sgRNA assembly by M13 and f1 G8P_PD_ prior to sgRNA addition. Cas9 to sgRNA ratio is fixed to 0.3. The above results (b-c) are shown as mean ± SD (*n* = 3).


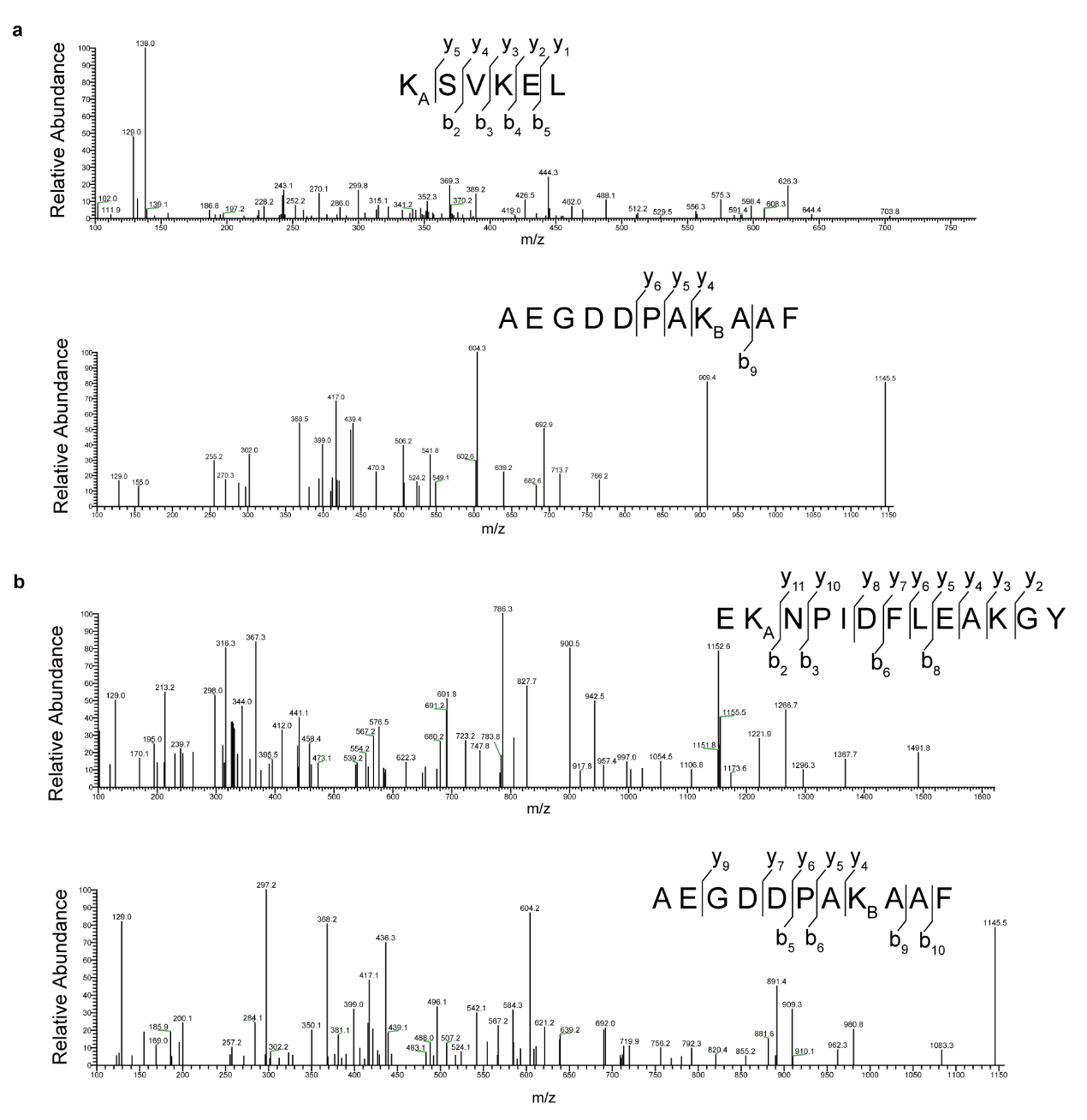


**Figure S2** MS analyses of the interface between SpCas9 and M13 G8P_PD_. (**a**) Tertiary MS for individual peptides KSVKEL and AEGDDPAKAAF as described in Fig. 3b. (**b**) Tertiary MS for individual peptides ENKPIDFLEAKGY and AEGDDPAKAAF as described in Fig. 3c.

**
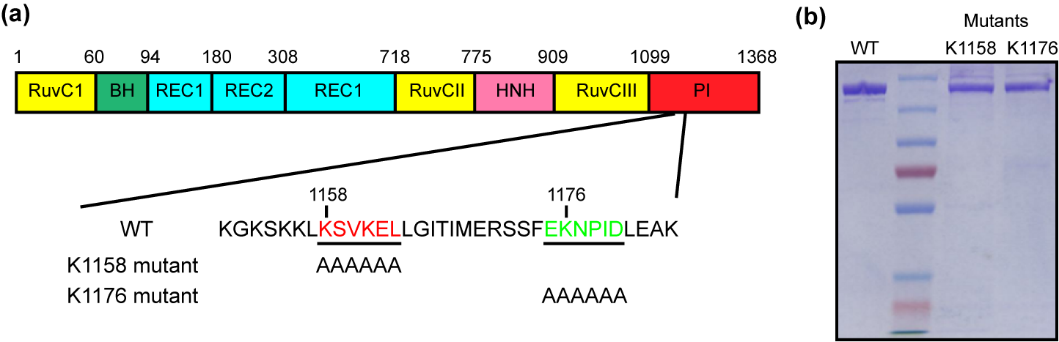
**

**Figure S3** Construction and purification of SpCas9 K1158 and K1176 mutants. (**a**) Schematic presentation of the structural organization of *S. pyogenes* Cas9. BH, bridging helix. PI, PAM interacting domain. Positions of alanine mutations in K1158 and K1176 mutants are indicated. (**b**) Purified WT, K1158 mutant and K1176 mutant SpCas9 proteins.


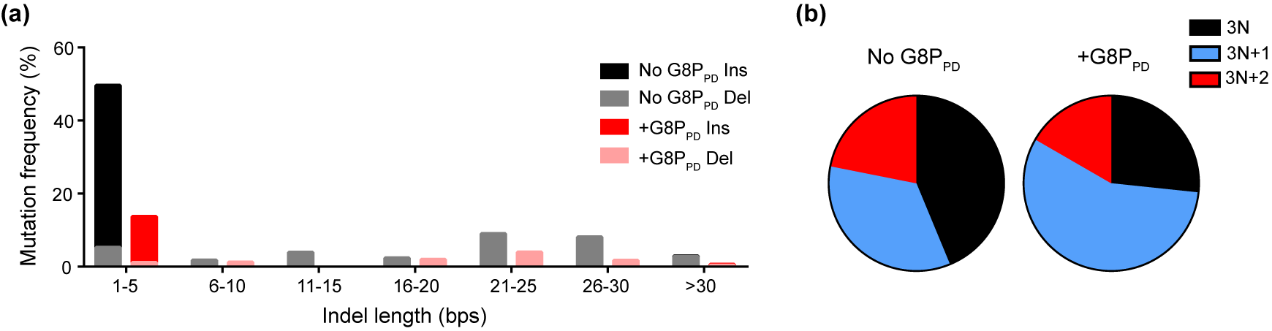


**Figure S4** Profile of SpCas9-induced mutations in the absence and presence of G8P_PD_. (**a**) Distribution of indel length. (**b**) Distribution of indel frame phase calculated as the length of indel modulus. The mean value of two biological replicates are shown.

**
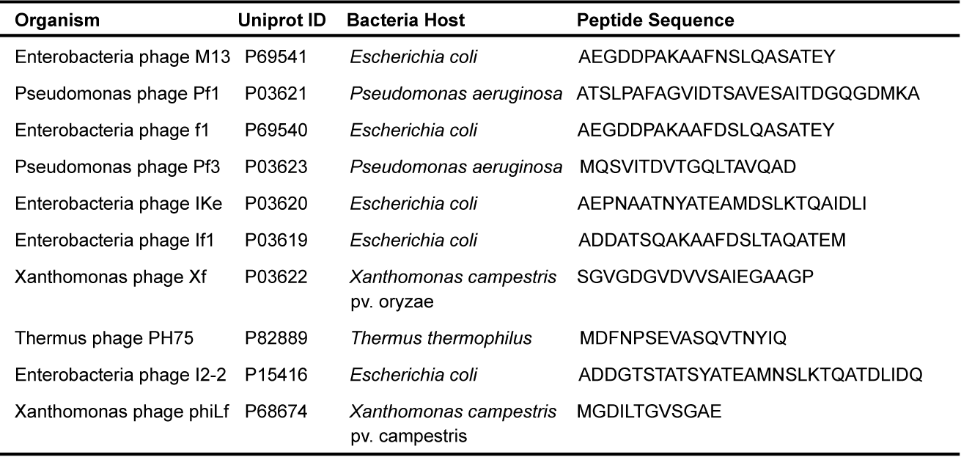
**

**Figure S5** G8P_PD_ peptides derived from inoviridae bacteriophages.


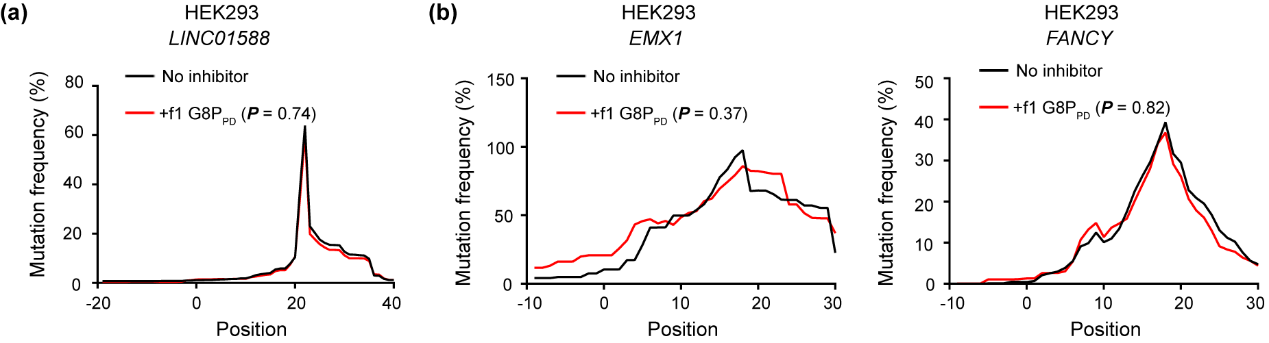


**Figure S6** NGS analyses of the effects of G8P pre-incubation on NmCas9 (a) and SaCas9 (b) in HEK293 cells. The mean value of three biological replicates are displayed. Significant difference between test group and mock is determined by two-tailed, unpaired Student’s *t* test. The adjusted *P* values are indicated.

**
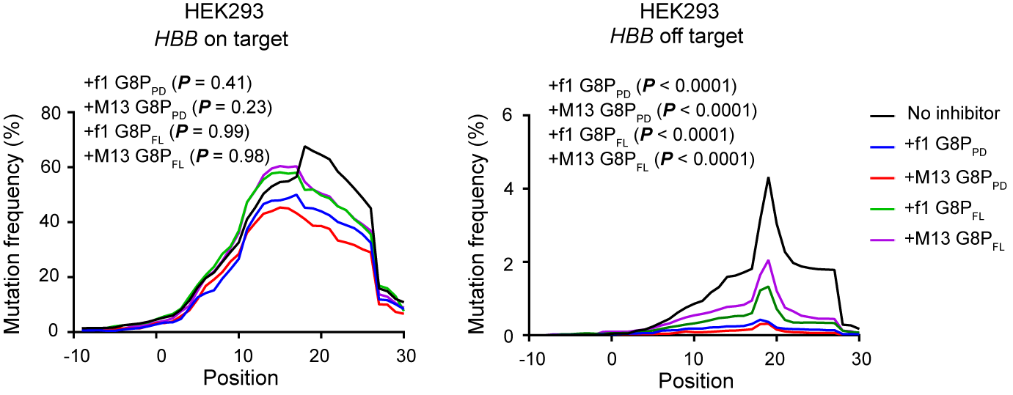
**

**Figure S7** NGS analyses of the effects of G8Ps on the specificity of SpCas9 at *HBB* site in HEK293 cells. The mean value of three biological replicates are displayed. Significant difference between test groups and mock is determined by one-way ANOVA with Dunnett’s multiple comparisons test. The adjusted *P* values are indicated.


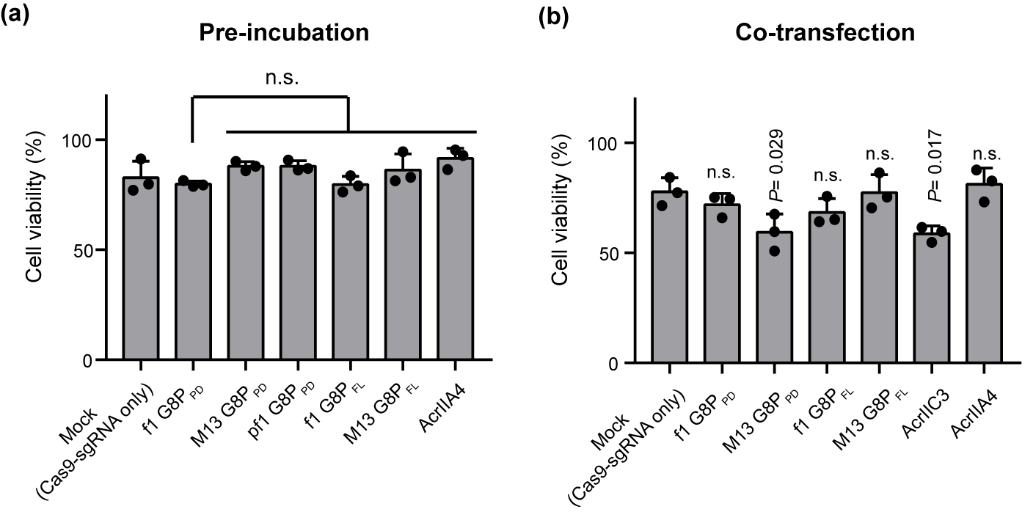


**Figure S8** Cytotoxicity of CRISPR inhibitors. Plasmids encoding CRISPR inhibitors are pre-incubated with or co-transfected in HEK293 cells as described in the Methods in the main text. Cell viability was determined using CCK-8 assay. The results are shown as mean ± SD (*n* = 3). Significant difference between test groups and mock is determined by one-way ANOVA with Dunnett’s multiple comparisons test. The adjusted *P* values are shown. n.s., no significant difference.


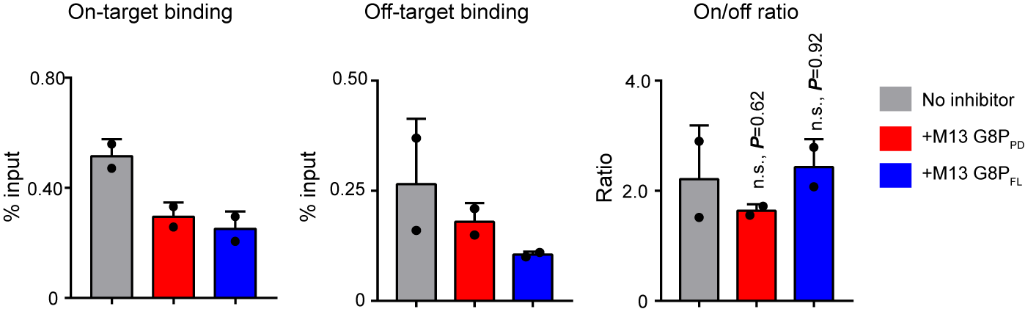


**Figure S9** ChIP-qPCR analyses of the effects of M13 G8Ps on Cas9 binding at *AAVS1* on target and pre-determined off-target sites in Hela cells. The results of two biological replicates are shown as mean ± SD. Significant difference between test groups and mock (no inhibitor) is determined by one-way ANOVA with Dunnett’s multiple comparisons test. The adjusted *P* values are shown. n.s., no significant difference.
