# Supplementary material for: Allosteric inhibition of CRISPR-Cas9 by bacteriophage-derived peptides
Source: Genome Biol. 2020 Feb 26;21:51. doi: 10.1186/s13059-020-01956-x (PMC7045643; doi:10.1186/s13059-020-01956-x)
Supplement: Supplementary file 3 — Review history. [file 13059_2020_1956_MOESM3_ESM.docx]

Review History

**First round of review**

**Reviewer 1**

**Are you able to assess all statistics in the manuscript, including the appropriateness of statistical tests used?**

Yes, and I have assessed the statistics in my report.

**Comments to author:**

Cui and Wang et al., report the discovery of new Cas9-inhibiting peptides, sourced from the phage major coat protein G8P (G8PPD), from inoviridae Bacteriophages. The authors suggest that this family of coat proteins, and a small internal peptide thereof, appear to function by disrupting Apo-SpyCas9's interaction with gRNAs. Overall the study is of high-quality and will be very useful to the field moving forward. I believe that the work is suitable for publication in Genome Biology pending a couple of key experiments and minor revisions to the text that will broaden impact and strengthen conclusions.

Key Experiments:
The major utility of these new peptides will likely be within human cells. Therefore, a more extensive analysis of common human cell targets is needed in figures 5 and 6. For example, VEGF, FANCA, RUNX are common targets used to verify off targeting and cleavage efficiency.

The authors need to demonstrate the specificity of these peptides for particular Cas9 orthologues. For example, do these peptides inhibit Sau/Nme/St Cas9? What about Cascade systems?

The authors should clarify the rationale to BLAST peptide homologs and why f1 and M13 peptides were subsequently used interchangeably. It would be important to derive the inhibitory concentrations/binding affinities between these two peptides to justify the interchangeability in addition to the sequence alignment shown in supplementary data. This will help future users determine which peptides will be optimal for their experiments.

The potential to mitigate off targets and maintain on target efficacy is potentially the most important use of this new technology. Mechanistically, however, this phenomenon is very confusion. Can the authors use ChIP-seq to show that binding is equivalent with and without inhibition?

Minor Revisions:
Generally, certain methods lack clarity which will obviously make repetition challenging. Specifically:
-The sequences of all in vitro and in vivo gRNAs should be listed in the methods section of the main text. The protospacer sequences and targets used in vitro are not clearly explained in the main text.
-Did the authors control for dual transfections in Figure 5 experiments? One could imagine serial transfections could increase cell stress when doing serial transfections. Doing this to one group and not the other may obfuscate results. An experimental design figure would be helpful, as the authors describe sorting for GFP+/mChe+ cells (presumably after receiving both plasmids) but don't really go into how this was all laid out in the methods or supplementary data.
-All expression plasmids should be deposited on Addgene to broaden impact of manuscript.

An important caveat to this new method of inhibition is the order-of-addition-dependence, one suggestion would be to state this in the abstract.

The authors should include a citation and more information regarding small molecule anti-CRISPRs to balance depth of coverage of the Acr systems and describe how/where they primarily.

None of the in vitro work included full length G8P yet it appears to be more effective at inhibiting gene modification than the periplasmic domain. Was this choice made because the PD was smaller? Can the authors explain and/or repeat same in vitro studies with the full length protein?

Line 7 page 6: change "relative" to "relatively'.

Figure 5 legend: "arrow denotes cleavage product" should be removed.

**Reviewer 2**

**Are you able to assess all statistics in the manuscript, including the appropriateness of statistical tests used?**

Yes, and I have assessed the statistics in my report.

**Comments to author:**

The manuscript presented by Cui et al is a well-written and timely research study. The authors explore alternative methods in which to inhibit Cas9 cleavage, which serves a critical unmet need as studies of mechanism and therapeutic efficacy begin to take prominence. While many other approaches are underway to specifically block or inhibit CRISPR/Cas9 activity, this is one of the first to follow the novel approach of bacteriophage-derived peptides. The manuscript lays out a series of experiments that are well-preformed with appropriate concerns and the discussion is supported by the results. The manuscript is clearly written and is novel. However, there are several minor concerns that should be addressed prior to publication.

Major concerns:
-The mechanism of G8Ppd on spCas9 inhibition is not clearly defined. The authors propose many mechanisms and perform experiments that seem to confirm specific theories, but then disprove themselves. Do they have a central hypothesis that they can focus the discussion and provide future perspectives on studies?
-The authors seem to contradict themselves on page 11 regarding the timeline in which G8Ppd functions on suppression. It seemed clear that G8Ppd had to be present prior to the formation of the RNP, however they suggest inhibition of the RNP when G8Ppd was added first. Are they suggesting that G8Ppd somehow limits binding and activity of the already formed RNP?
- On page 12 the authors suggest that G8Ppd can serve as a "safe off-switch". However at the top of page 12 and on page 11, I am led to believe that G8Ppd will only be effective if the peptide is added prior to the formation of the RNP complex. If this is the case, wouldn't this compound only serve as an inhibitor blocking the effect, not capable of "turning off" Cas9 activity?
-The text on the bottom of page 13 is unclear.
- Page 15 the authors again seem to contradict themselves on the function of G8Ppd. Is it possible for the authors to explore the confrontational change they are suggesting to Cas9 following non-competitive binding to G8Ppd?
- One limiting factor of the overall use of G8Ppd in this manuscript as it applies to the CRISPR field as it continues to advance is the applicability of this peptide to new Cas variants. Many groups are evaluating other naturally occurring variants in addition to directly evolving the protein, would the authors speculate that this peptide would function across multiple Cas variants.

Minor concerns:
- The Cas9 is typically reported as spCas9 not spyCas9.
- Page 6 Lines 1-9 is lacking citations.
- Page 8 Line 25-26 apo-spCas9 is not defined previously
-Page 9 line 15: can the authors define what they mean by weak interaction?
-Page 9 line 23: define "0.3" molar ratio

**Authors Response**

**Point-by-point responses to the reviewers’ comments:**

Reviewer reports:

Reviewer #1: Cui and Wang et al., report the discovery of new Cas9-inhibiting peptides, sourced from the phage major coat protein G8P (G8PPD), from inoviridae Bacteriophages. The authors suggest that this family of coat proteins, and a small internal peptide thereof, appear to function by disrupting Apo-SpyCas9's interaction with gRNAs. Overall the study is of high-quality and will be very useful to the field moving forward. I believe that the work is suitable for publication in Genome Biology pending a couple of key experiments and minor revisions to the text that will broaden impact and strengthen conclusions.

Key Experiments:

The major utility of these new peptides will likely be within human cells. Therefore, a more extensive analysis of common human cell targets is needed in figures 5 and 6. For example, VEGF, FANCA, RUNX are common targets used to verify off targeting and cleavage efficiency.

*We have examined the effects of G8P on the CRISPR-Cas9 activity at additional genomic site (Additional file 1: Figure S7). Consistent with the results at AAVS1 site, G8P can reduce the off-target activity of SpCas9 with little perturbation of its on-target activity at HBB.*

The authors need to demonstrate the specificity of these peptides for particular Cas9 orthologues. For example, do these peptides inhibit Sau/Nme/St Cas9? What about Cascade systems?

*We thank the reviewer for this helpful comment. We have investigated the effects of G8P on SaCas9 and NmCas9 and have found that overexpressed G8P did not inhibit SaCas9 or NmCas9 at the examined genomic sites in human cells (Additional file 1: Figure S6). It will be also interesting to investigate the effects of G8P on Cascade systems and we intend to initiate an in-depth study in a separate study.*

The authors should clarify the rationale to BLAST peptide homologs and why f1 and M13 peptides were subsequently used interchangeably. It would be important to derive the inhibitory concentrations/binding affinities between these two peptides to justify the interchangeability in addition to the sequence alignment shown in supplementary data. This will help future users determine which peptides will be optimal for their experiments.

*We have revised the main text to better clarify the design and rationale of our experiments. A direct comparison of f1 and M13 G8PPD is shown (Additional file 1: Figure S1a-c). We found that f1 G8P has better aqueous solubility than M13 G8P. Given the similar inhibiting activities, f1 and M13 G8Ps were used interchangeably to rule out potential artifact due to the solubility issue, especially in the experiments that require high concentrations of peptides.*

The potential to mitigate off targets and maintain on target efficacy is potentially the most important use of this new technology. Mechanistically, however, this phenomenon is very confusion. Can the authors use ChIP-seq to show that binding is equivalent with and without inhibition?

*A ChIP-qPCR experiment has been performed to determine the binding of catalytically inactive Cas9 at the AAVS1 on- and off-target sites in the presence or absence of G8P (Additional file 1: Figure S9). It appeared that the presence of G8P did not significantly improve the specificity of binding at on-target site, suggesting that other mechanisms may involve in G8P-mediated improvement of targeting specificity of SpCas9 at AAVS1 site.*

*Previous study has shown that excess Cas9 protein from constitutive expression will lead to increased off-target cleavage without further increasing the on-target cleavage due to saturated activity (Hsu et al., 2013, Nat Biotech). We hypothesize that the presence of G8P may constrain the activity of excess Cas9 proteins. The Discussion has been revised and a cartoon provided to better rationalize our hypothesis (Figure 7). We admitted, however, that though our observation aligns with previous theories, a more careful study is needed to determine the detailed mechanism of action of G8P in living cells.*

Minor Revisions:

Generally, certain methods lack clarity which will obviously make repetition challenging. Specifically:

-The sequences of all in vitro and in vivo gRNAs should be listed in the methods section of the main text. The protospacer sequences and targets used in vitro are not clearly explained in the main text.

*We have provided sgRNA sequences in the Methods of the revised manuscript.*

-Did the authors control for dual transfections in Figure 5 experiments? One could imagine serial transfections could increase cell stress when doing serial transfections. Doing this to one group and not the other may obfuscate results. An experimental design figure would be helpful, as the authors describe sorting for GFP+/mChe+ cells (presumably after receiving both plasmids) but don't really go into how this was all laid out in the methods or supplementary data.

*We apologize for not clarifying this important experimental procedures and thank the reviewer to point this out. Yes, the experiments in Figure 5 are well controlled and detailed information has been included in the Methods of the revised manuscript. Briefly, sgRNA and G8P plasmids contain GFP and mCherry reporters respectively. The mock groups without G8P expression were transfected with an empty mCherry-expressing plasmid to control for cell stress. An experimental design figure has been included (Fig. 5a). In addition, we have determined the cell viability under G8P pre-incubation and co-transfection conditions to rule out the effects of cytotoxicity (Additional file 1: Figure S8) and the text has been revised accordingly.*

-All expression plasmids should be deposited on Addgene to broaden impact of manuscript.

*We have initiated the deposition process and the information for all deposited plasmids will be included in the final version of this manuscript.*

An important caveat to this new method of inhibition is the order-of-addition-dependence, one suggestion would be to state this in the abstract.

*We have revised the Abstract to describe this particular feature of G8P.*

The authors should include a citation and more information regarding small molecule anti-CRISPRs to balance depth of coverage of the Acr systems and describe how/where they primarily.

*We apologize for missing the citation for small molecule anti-CRISPRs in Introduction. We have added the citation along with more information for these small molecule CRISPR inhibitors (Maji et al, 2019, Cell).*

None of the in vitro work included full length G8P yet it appears to be more effective at inhibiting gene modification than the periplasmic domain. Was this choice made because the PD was smaller? Can the authors explain and/or repeat same in vitro studies with the full length protein?

*We attempted to express and purify a His-tagged full-length G8P from E. coli using pET28a-BL21(DE) expression system but failed to collect adequate amount for in vitro analyses. One possible reason is that the coat protein G8P contains a transmembrane helix (position 45-65), causing folding problem of the expressed protein cargos. We have included a statement in the Methods to provide this information.*

Line 7 page 6: change "relative" to "relatively'.

*This typo has been corrected.*

Figure 5 legend: "arrow denotes cleavage product" should be removed.

*This typo has been corrected.*

Reviewer #2: The manuscript presented by Cui et al is a well-written and timely research study. The authors explore alternative methods in which to inhibit Cas9 cleavage, which serves a critical unmet need as studies of mechanism and therapeutic efficacy begin to take prominence. While many other approaches are underway to specifically block or inhibit CRISPR/Cas9 activity, this is one of the first to follow the novel approach of bacteriophage-derived peptides. The manuscript lays out a series of experiments that are well-preformed with appropriate concerns and the discussion is supported by the results. The manuscript is clearly written and is novel. However, there are several minor concerns that should be addressed prior to publication.

Major concerns:

-The mechanism of G8Ppd on spCas9 inhibition is not clearly defined. The authors propose many mechanisms and perform experiments that seem to confirm specific theories, but then disprove themselves. Do they have a central hypothesis that they can focus the discussion and provide future perspectives on studies?

*We have extensively revised the Results and Discussion and provided a cartoon (Fig. 7) to better illustrate our hypothesis and to provide perspectives for future studies.*

-The authors seem to contradict themselves on page 11 regarding the timeline in which G8Ppd functions on suppression. It seemed clear that G8Ppd had to be present prior to the formation of the RNP, however they suggest inhibition of the RNP when G8Ppd was added first. Are they suggesting that G8Ppd somehow limits binding and activity of the already formed RNP?

*We have included a flowchart to explain the experimental design of our experiments (Fig. 5a). The Cas9 and sgRNA involved in Fig. 5a are overexpressed from mammalian expression plasmids. The in vitro data suggest that G8PPD can suppress Cas9-sgRNA binding only at high concentrations and that the interaction between Cas9 and G8PPD is relatively weak compared to that between Cas9 and sgRNA. Hence, we pre-incubated cells with G8P-expressing plasmid such that G8P at saturated concentrations can block the assembly of subsequently transfected Cas9 and sgRNA.*

- On page 12 the authors suggest that G8Ppd can serve as a "safe off-switch". However at the top of page 12 and on page 11, I am led to believe that G8Ppd will only be effective if the peptide is added prior to the formation of the RNP complex. If this is the case, wouldn't this compound only serve as an inhibitor blocking the effect, not capable of "turning off" Cas9 activity?

*We agree with the reviewer that G8PPD block RNP assembly but not Cas9 cleavage activity. From a more general perspective, the inhibitors involving in any step of CRISPR-Cas9 function (sgRNA binding, DNA binding, DNA cleavage etc.) are deemed as CRISPR off-switches (Pawluk et al., 2018, Nat Rev Microbiol; Stanley et al., 2018, Annu Rev Genet). We adopt this general concept of CRISPR off-switch in the present study.*

*-*The text on the bottom of page 13 is unclear.

*We have revised the text for enhanced quality.*

- Page 15 the authors again seem to contradict themselves on the function of G8Ppd. Is it possible for the authors to explore the confrontational change they are suggesting to Cas9 following non-competitive binding to G8Ppd?

*We have revised the text to better illustrate our hypothesis. In-depth characterization of the mechanism of G8P is certainly an interesting topic that we would like to pursue in future studies, but it may be out of the scope of the present study that is focused on evaluating the biological activity of G8P.*

- One limiting factor of the overall use of G8Ppd in this manuscript as it applies to the CRISPR field as it continues to advance is the applicability of this peptide to new Cas variants. Many groups are evaluating other naturally occurring variants in addition to directly evolving the protein, would the authors speculate that this peptide would function across multiple Cas variants.

*We thank the reviewer for this helpful comments. We have examined the effects of G8Ps on SaCas9 and NmCas9 but observed no significant inhibition in human cells (Additional file1: Figure S6).*

Minor concerns:

- The Cas9 is typically reported as spCas9 not spyCas9.

*We have changed SpyCas9 to SpCas9 in the revised manuscript.*

- Page 6 Lines 1-9 is lacking citations.

*We thank the reviewer to point this out and have added appropriate citations.*

- Page 8 Line 25-26 apo-spCas9 is not defined previously

*We have defined the apo-SpCas9 in the main text.*

-Page 9 line 15: can the authors define what they mean by weak interaction?

*We have revised the text to clarify this.*

-Page 9 line 23: define "0.3" molar ratio

*We have revised the text to clarify this.*

**Second round of review**

**Reviewer 1**

Cui et al. have done a great job of responding to all of my previous concerns. The manuscript "Allosteric Inhibition of CRISPR-Cas9 by Bacteriophage-derived Peptides" is now suitable for publication. The increased clarity, explanations, and data (provided in both the main text and extensive new supplemental info) strengthen the impact and utility of the findings.

**Reviewer 2**

The authors appropriately addressed all my concerns.
